# Quantifying and Controlling DNA Probe Density on the Surface of Silicon Nitride Optical Waveguides

**DOI:** 10.1021/acs.langmuir.5c01064

**Published:** 2025-04-23

**Authors:** Samer Aphrham, Mark Verheijden, Jurriaan Huskens

**Affiliations:** † Department of Molecules and Materials, Faculty of Science & Technology, MESA+ Institute and TechMed Centre, University of Twente, PO Box 217, Enschede 7500 AE, The Netherlands; ‡ Qurin Diagnostics B.V, Emmy Noetherweg 2, Leiden 2333 BK, The Netherlands

## Abstract

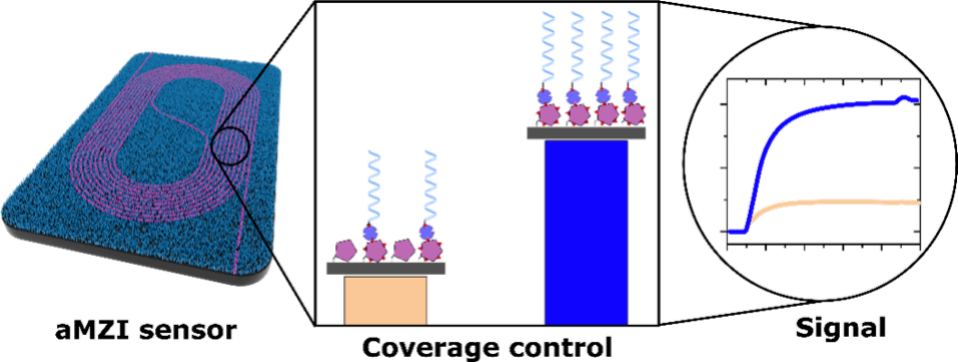

Photonic biosensors offer a label-free, sensitive, and cost-effective means of detecting pathogens and biomarkers, such as methylated DNA, in liquid biopsy samples. However, challenges persist in controlling and quantifying the surface density of probes and complementary targets, which is essential to achieve optimal sensitivity. To address these issues in DNA detection, the surfaces of asymmetric Mach–Zehnder interferometer (aMZI) waveguide sensors were functionalized using two approaches to achieve density-controlled probe-DNA surfaces. In one method, varying ratios of BSA and biotinylated BSA were incubated on each sensor surface, followed by neutravidin and biotinylated probe DNA (pDNA), allowing for controlled surface coverage on each aMZI sensor. A second approach involved direct binding of amino-pDNA, mixed with nonprobe DNA, to the carboxylated aMZI surface after EDC-NHS activation. Target-DNA (tDNA) hybridization was then introduced at different concentrations to assess the effect of surface density on binding. A quantification method was developed to account for the molecular mass density, enabling the estimation of real-time signal responses during both protein functionalization and DNA binding steps. Results showed that higher tDNA solution concentrations exhibited a strong dependence on surface coverage, while lower concentrations showed a minimal dependence. Fluorescence spectroscopy, using fluorescently labeled tDNA, confirmed a direct linear correlation between the surface density and fluorescence intensity, offering a simpler yet robust method for quantitative surface characterization. This correlation provides an alternative method for estimating surface density without the need for laborious characterization. This study contributes to the development and understanding of photonic biosensing techniques for biomarker detection in liquid biopsy samples.

## Introduction

According to the 2020 report from the World Health Organization (WHO), nearly 75% of the approximately 20.4 million premature deaths (occurring between the ages of 20 to 70) are attributed solely to noncommunicable diseases.^1^ Such diseases encompass chronic and slow-progressing medical conditions, with cardiovascular diseases, diabetes, and cancer being the most prevalent among them. Moreover, three out of every ten individuals suffering from noncommunicable diseases succumb to cancer.^1^ The increasing global cancer burden highlights the urgent need for technological advancements to enhance early detection. Integrating genomic knowledge and data science offers significant potential for improving the accuracy of diagnosis and detecting cancers at earlier stages. Screening, aimed at identifying cancer in individuals without symptoms, is crucial for early intervention and improved survival rates. However, despite efforts, nearly half of all cancer cases are diagnosed at advanced stages.^2^ Key challenges include identifying, validating and detecting biomarkers amidst physiological noise at extremely low biomarker quantities and prioritizing minimally invasive sampling methods like liquid biopsies (i.e., saliva, blood, and urine).^2,3^ Innovative technologies such as nanobiotechnology and optical biorecognition play pivotal roles in enabling highly sensitive diagnoses and personalized treatments, crucial for combating cancer effectively.^4^ In the field of cancer diagnostics, biosensing technologies offer noninvasive, point-of-care (PoC) and sensitive approaches for early detection and personalized treatment.^5,6^

Biosensors represent a powerful class of analytical devices that integrate biological recognition elements with transducers to detect and quantify specific analytes. These versatile tools have the potential to revolutionize various fields, from medical diagnostics to environmental monitoring, by providing rapid, sensitive, and selective detection capabilities.^7^ At the heart of biosensors lies their ability to convert biological interactions into measurable signals, making them invaluable for understanding complex biological processes and facilitating diagnostic and therapeutic interventions.

Many surface-based biosensing techniques rely on molecular labeling to create a signal. Labeling can potentially alter the function of the receptor-analyte interaction including sensitivity and selectivity. For example, the addition of fluorescent or redox-active labels may interfere with the binding affinity, conformation, or activity of the biomolecular target being studied. In some cases, this alteration can affect the accuracy and relevance of the biosensing results, particularly in studies of delicate biological processes.^7,8^ As an alternative to labeled biosensing, label-free biosensing technologies have been developed that overcome these limitations.^8^ Furthermore, label-free biosensing offers advantages in terms of simplicity and cost-effectiveness. Finally, it gives direct information on the biomolecule’s binding behavior, such as the binding kinetics.^9^ Examples of label-free biosensing techniques include surface plasmon resonance (SPR), quartz crystal microbalancing (QCM), and impedance spectroscopy. The widespread adoption of these technologies is still hindered by issues related to sensitivity, scalability, multiplexing capabilities, and the absence of internal references for complex matrices.

A promising solution could be the emerging photonic sensor based on the asymmetric Mach–Zehnder interferometer (aMZI), which may address these challenges.^9−12^ The aMZI exploits the optical phenomenon of the propagating evanescent field to monitor biomolecular interactions in real-time. When target molecules bind to the sensor surface, they induce changes in the refractive index (RI), which can be measured as phase shifts in the aMZI signal. As a result of the feasibility of detecting such phase shifts with very high accuracy, the aMZI shows excellent sensitivity. Notably, It is well established that the optical signal generated by refractive index (RI) changes directly corresponds to absolute molecular density, as demonstrated in techniques like ellipsometry and surface plasmon resonance spectroscopy.^8,13,14^ Moreover, optical chip manufacturing is scalable, which reduces fabrication costs and facilitates the application in the medical diagnostics field, potentially as point-of-care devices.^15^

Currently, aMZI-based biosensing lacks a proper quantification method suitable for off-line probe immobilization needed for multiplexing, as well as wafer-scale production. In addition, the correction between the optical signal and the surface density of probes and targets was shown in literature for proteins;^16,17^ however, it remains undefined for most biocomponents and especially of DNA. Moreover, most studies focus on quantification at the higher probe density regime. We expect that by carefully controlling the probe density on the surface and using the highly sensitive aMZI sensors, more insight on surface quantification can be obtained, which can further assist in detecting variability or patterns in binding efficiency that might otherwise be overlooked. Such knowledge may become important to facilitate the detection of ultralow concentrations of biomarkers.

In this work, we aim to introduce a method to control the density of single-strand DNA (ssDNA) probes on the aMZI waveguide surface and to quantify the aMZI signal response to binding complementary target DNA (tDNA). We assess the surface densities of probes and complementary targets using different probe immobilization chemistries, and we aim to correlate the signal response to fluorescence intensity using labeled probes or targets as a second independent measure of surface density. In the following sections the instrumentation and experimental setup are presented, followed by a strategy for controllable protein and DNA probe surface functionalization. Subsequently, a detailed description of the quantification method used for the density estimation of the hybridized probe and tDNA is provided, and finally, the relation between fluorescence intensity to the surface density is established and compared to aMZI responses.

## Results and Discussion

### Sensor Setup and Surface Functionalization Strategy with Probe Density Control

**Figure 1:**
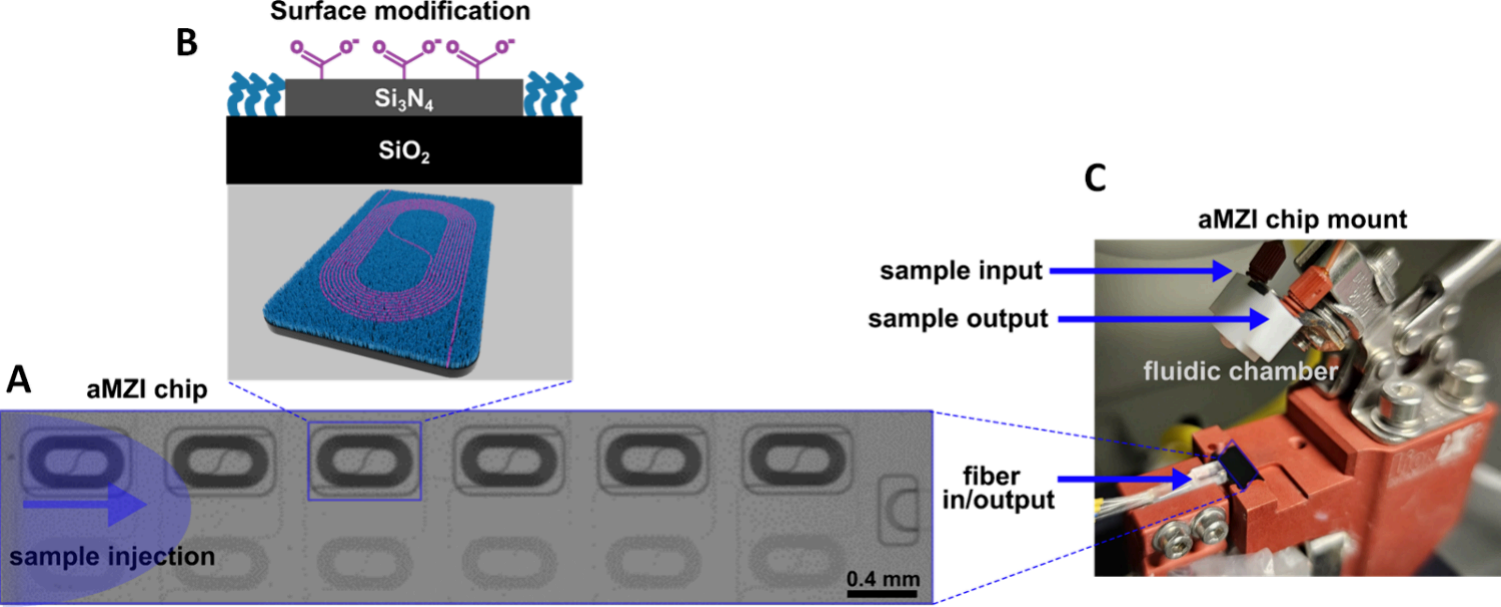
Pictures showing the experimental setup: (A) brightfield image of a single aMZI chip with six individual aMZI sensors including the sensor arm with the exposed “dark” spiral-shaped waveguide (top) and the reference arm covered with SiO_2_ cladding (bottom). (B) Schematic cross-section and 3D illustration of a sensor arm depicting the material-selective coating with carboxylic acid on the waveguide (magenta) and the surrounding PEG-based antifouling layer (blue). (C) The aMZI chip mount contained an in/out fiber interface connected to an aMZI chip. The mount is integrated with a fluidic chamber, which is clamped onto the chip during real-time measurement to provide a continuous flow of samples and a running buffer.

The TriPleX photonic waveguide chips utilized in this study contain six individual aMZI sensing elements on each chip;^11,12,18^ see Figure 1A. The chip surface is protected with a layer of SiO_2_ cladding, with specific sections of the cladding etched away to expose the Si_3_N_4_ waveguides, thus facilitating interaction with the surrounding environment. Each sensor element operates independently and is monitored separately, enabling simultaneous measurements of all six sensors. A single fluidic chamber is affixed to the chip, serving to introduce samples of interest across the entire chip surface, as depicted in Figure 1C. During real-time measurement, the chip surface is preconditioned by continuously flowing a buffer (running buffer) followed by a sample fluid injection into the fluidic chamber. This sample fluid gradually fills the chamber and traverses across the chip surface, allowing for the interaction with the exposed waveguide on each sensor. Eventually this process facilitates controlled data acquisition and multiplexing of functionalized analytes on the chip surface. In order to increase the selectivity of the aMZI sensor, the surface is treated with a commercially available, material-selective coating, consisting of a thin organic layer with terminal carboxylic acid groups on the Si_3_N_4_ waveguide and a PEG-based antifouling layer on the remaining SiO_2_ surface area (the nonsensing area);^19^ see Figure 1B. Consequently, the sensing areas can be selectively modified with a biorecognition layer, leaving the surrounding SiO_2_ area unfunctionalized.

In order to obtain a density-controlled biorecognition layer, initially a reliable surface modification method has been adopted based on the well-known biotin-neutravidin (NAv) binding motif. The surface modification starts with an activation step using conventional EDC/NHS chemistry, which functionalizes the carboxylic acid groups on the Si_3_N_4_ waveguide with an amine-reactive NHS-ester. Hereafter, the ester is allowed to react with free amines of the bovine serum albumin (BSA) protein, producing an antifouling layer on the surface. Besides regular BSA, BSA decorated with biotin moieties was utilized to establish a secondary layer using NAv binding, to which biotin-modified receptors can be bound. The high specificity and affinity of NAv (and of its partner protein streptavidin, SAv) for biotin is one of most exploited noncovalent complexes in many techniques.^20,21^

**Figure 2:**
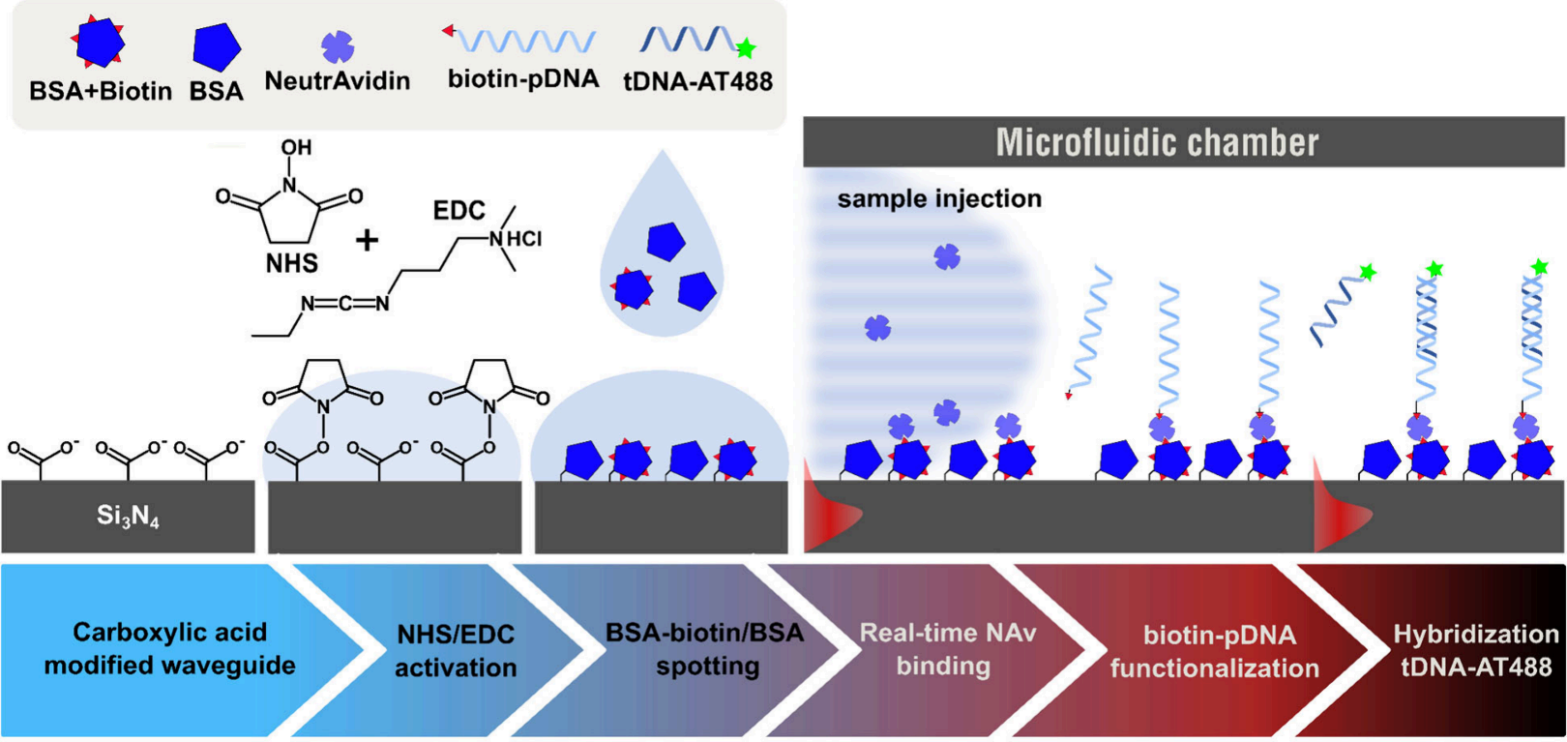
Schematic illustration of NHS/EDC surface activation followed by the spotting of different mixtures of BSA-biotin/BSA on each aMZI sensor to create controllable surface functionalization. Hereafter the chip was washed and placed in a flow chamber, and NAv was allowed to flow and bind to the BSA-biotin. The binding events were monitored in real-time, followed by adsorption of biotinylated ssDNA probe (biotin-pDNA) and finally the hybridization of its complementary ssDNA target stand (tDNA-AT488). Schematics are not to scale.

To achieve receptor density control, a set of solutions containing biotin-functionalized BSA (BSA-biotin) and unmodified BSA at different ratios were prepared and spotted on each individual aMZI sensor, as illustrated in Figure S1 (see Materials and Methods for more details). Consequently, precise densities of BSA-biotin were achieved on the waveguides. To follow the binding event of NAv in real-time, the modified chip was placed in a closed microfluidic chamber and the immobilization of NAv on the aMZI surface was measured *in situ*. The presence of unused biotin-binding pockets on the NAv protein, conveniently oriented to the sample solution, allows for the formation of a DNA biorecognition layer on top of the NAv-coated surface. Hereto, a biotinylated ssDNA probe (biotin-pDNA) was introduced into the microfluidic chamber and allowed to attach to the NAv layer (Figure 2). Finally, the hybridization of the complementary and fluorescently labeled ssDNA target sequence (tDNA-AT488) to the biotin-pDNA was measured in real-time, and fluorescence images were taken afterward. In this case, the fluorescence dye was added to the tDNA solely for further examination of the correlation between surface density, aMZI response, and fluorescence intensity, as explained above. It is not required or used for aMZI signal generation.

**Figure 3:**
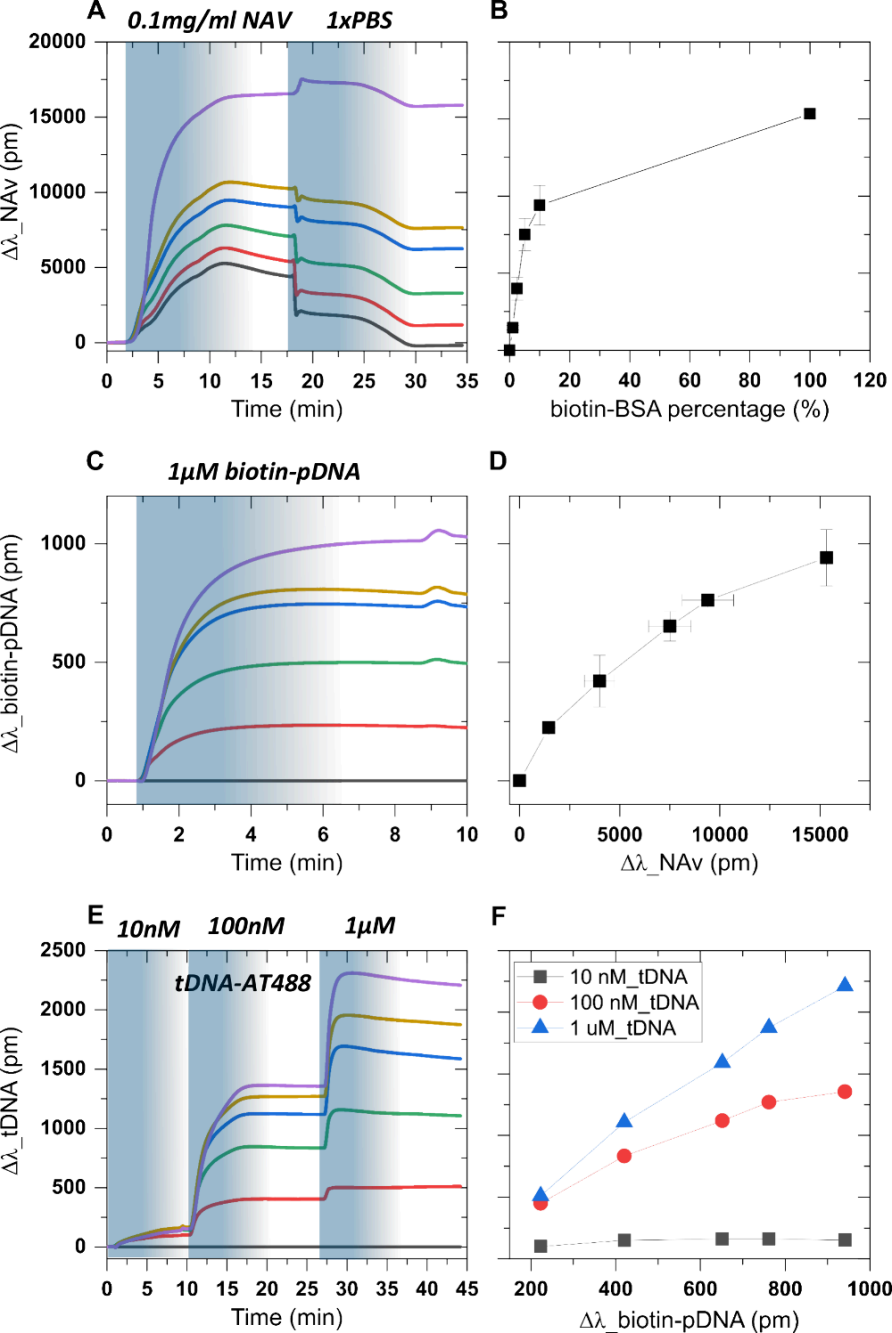
Functionalization of the six sensors of an aMZI chip with different densities of NAv and binding of biotinylated DNA probes and target DNA: Colored-line graphs depict the real-time response of six aMZI sensors coated with BSA/BSA-biotin upon introducing sample plugs: (A) 0.1 mg/mL NAv followed by a washing step with 1x PBS. (B) Obtained NAv signal response at saturation levels vs spotted fraction of biotinylated BSA. (C) Injection of 1 μM biotin-pDNA onto the NAv layer. (D) Probe DNA signal response vs the NAv signal. (E) Successive injections of different concentrations (10 nM, 100 nM, and 1 μM) of tDNA-AT488 onto the NAv and probe-DNA-functionalized sensor surfaces. (F) tDNA signal responses vs biotin-pDNA signals obtained on each individual aMZI sensor. All injections were obtained under constant flow of designated running buffer (see Materials and Methods).

The real-time measurement is obtained by continuously injecting a running buffer into the microfluidic chamber containing the aMZI biosensor, while short sample injections of NAv, probe, and target are performed (for more information on the injection process see Materials and Methods). The aMZI sensor responses to the binding steps during the injection of NAv, pDNA, and tDNA are listed in Figure 3. The response by each individual aMZI sensor is depicted by colored lines, and the duration of each sample injection is indicated by blue gradient areas, representing the axial mixing at the end of the sample plug (Materials and Methods). To establish a stable baseline, continuous infusion of running buffer is employed prior to each sample injection. For each functionalization step an optimized running buffer was selected to ensure maximal binding efficiency.

The surface functionalization starts with an injection of a 1.67 nM (0.1 μg/mL) NAv sample into the flow chamber, resulting in a swift signal increase indicative of NAv binding to each individual aMZI sensor (Figure 3A). Notably, aMZI-6 (depicted in purple), which has been spotted with the highest fraction of biotin-BSA, exhibited a maximal signal shift, whereas aMZI-1, which was functionalized with regular BSA only, registered the lowest response. After a 10 min interval, equilibrium was achieved. However, in short succession, weakly bound, physisorbed NAv gradually dissociated from the surface, leading to a gradual decline in signal attributed to a concentration gradient at the end of the sample plug due to axial mixing.^22^ To expedite the dissociation process, PBS was injected, resulting in a sharp reduction in the signal around the 18 min mark. The observed reduction here is due to the change in the net charge of NAv (pI 6) from positive (10 mM MES pH 5.5) to negative (PBS pH 7.4), promoting the rapid dissociation of weakly bound NAv from the surface. Subsequently, the end of the sample plug (PBS) was reached, reintroducing running buffer (MES) into the flow chamber. At the end of the functionalization process (at ∼30 min), the chip surface exhibited a stable response. The obtained total signal shift due to NAv binding at saturation levels aligns well with previously incubated BSA/BSA-biotin fractions on each sensor surface (Figure 3B), confirming successful control over surface density. Notably, the surface coverage displays a highly nonlinear mixed fraction of BSA and BSA-biotin, such that the biotinylated BSA protein shows a significantly higher affinity toward the surface compared to pure BSA. We speculate that the increase in affinity is attributed to the relatively less positively charged surface of biotinylated BSA compared to that of unmodified BSA. Consequently, the unmodified BSA may experience more electrostatic repulsion between proteins and/or the interface which limits its affinity with or approach to the surface. Additionally, the positively charged BSA protein may undergo “unfavorable” conformational arrangements, leading to a less densely packed protein coating.^23,24^ An alternative explanation for the observed nonlinear character of the NAv signal on the BSA surface is that the size of the NAv causes steric hindrance already at rather low biotin-BSA fractions, an effect that is often observed with SAv.^25^ Nevertheless, the maximal signal shift is observed on aMZI-6, indicative of maximal coverage on the aMZI surface due to incubation with 100% biotinylated BSA. For aMZI 2–5, the total generated signal on each aMZI successively increases. In contrast, the gradual decrease in the signal from aMZI-1 during washing, ultimately reverting to baseline levels, indicates the absence of NAv binding, suggesting the effectiveness of the BSA layer as an antifouling layer and making aMZI-1 a suitable negative control for subsequent surface functionalization. In conclusion, by spotting, using droplets with BSA and BSA-biotin in varying ratios, the complete coverage range from zero to full occupancy can be achieved and the NAv signal can be used as a benchmark to quantify the probe density on each sensor upon consecutive biotin-probe functionalization in the next step.

Similarly, the functionalization of the NAv-modified aMZI surface with biotin-pDNA was performed by sample plug injections using a solution of 1 μM biotin-pDNA. The signal response for aMZI-1 was subtracted from all data series, yielding the responses shown in Figure 3C (raw data shown in Figure S2A), to distinguish the binding attributable to specific biotin-NAv interactions. A swift increase in signal response was observed for aMZI-2–5 followed by leveling off before the end of the biotin-pDNA plug, indicating the saturation of the available NAv sites, which is expected at this probe concentration. The saturation response remains stable even after reintroducing the running buffer suggesting no dissociation from the surface, in agreement with the formation of strong NAv-biotin bonds. Furthermore, the biofunctionalization of biotin-pDNA on the NAv layer, plotted vs the signal of the preceding NAv binding step, shows a slowly saturating trend as shown in Figure 3D. This trend could be attributed to the increase in electrostatic repulsion between the biotin-pDNA probes at higher surface densities.

Finally, the hybridization of target DNA, tDNA-AT488, to the biotin-pDNA-functionalized surface was achieved by gradually increasing the concentration (from 10 nM to 1 μM) of the target in the sample plugs introduced onto the surface, resulting in steplike binding curves seen in Figure 3E (raw data shown in Figure S2B). Figure 3F shows the saturation signal values for the target as a function of the probe DNA signals observed in the preceding step. At a low (10 nM) tDNA concentration, the difference in probe coverage on each aMZI appeared to have a minimal effect on tDNA hybridization. However, at higher concentrations (100 nM, 1 μM), the effect of probe coverage becomes more noticeable (Figure 3F). We attribute the slow and comparable binding at low tDNA concentrations to mass transfer limitation, whereby the diffusion rate of the tDNA is rate-limiting instead of its hybridization on the surface, possibly assisted by electrostatic repulsion. At such a mass transport-limited regime, the hybridization rate is mostly dependent on the bulk concentration of analyte introduced into the flow chamber.^26,27^ At a high tDNA concentration (1 μM), diffusion is fast, and the pool of pDNA at the surface becomes rate-limiting, resulting in concentration-dependent hybridization (Figure 3F, blue line).

Fluorescence-based experiments were performed on the controlled BSA/BSA-biotin surface coverage previously presented in Figure 3. Upon forming a NAv layer on the BSA/BSA-biotin scaffold, an ssDNA-based recognition layer was created by injecting biotin-pDNA onto the surface. Subsequently, real-time hybridization of its target DNA (tDNA-AT488) was performed. Hereafter, we acquired the fluorescence emission of the AT488 dye conjugated to tDNA on each individual aMZI sensor. All acquired data were obtained using strictly fixed microscope settings as described in more detail in the Materials and Methods section.

**Figure 4:**
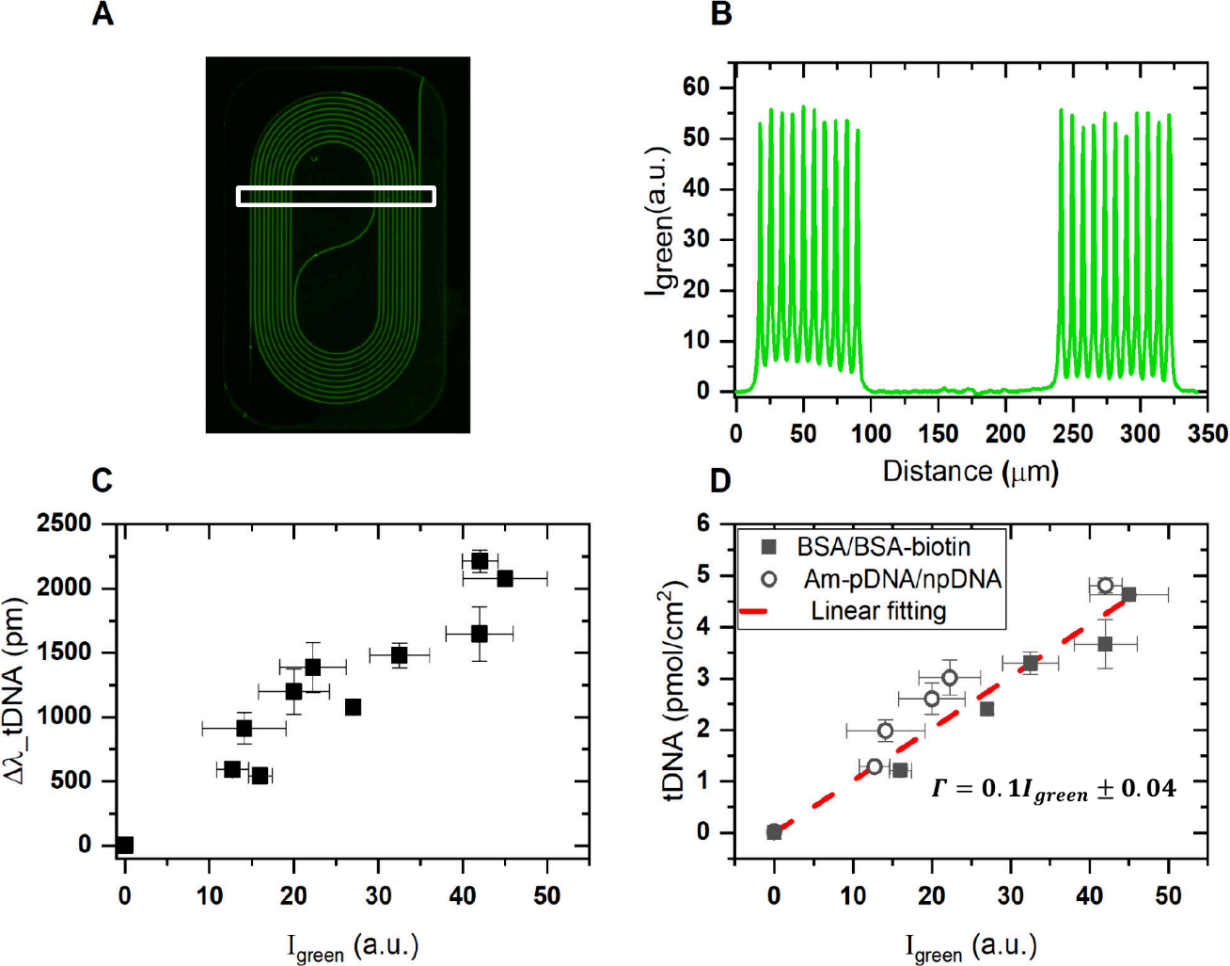
Fluorescence intensity quantification on the Si_3_N_4_ waveguide using (A) the fluorescence emission of Alexa 488-labeled tDNA upon hybridization on the aMZI waveguide surface prefunctionalized with biotin-BSA and NAv. (B) Peak intensity of fluorescence image obtained by selecting an area represented as white rectangle. (C) Collected fluorescence intensity from all aMZI measurements vs its real-time aMZI response at complete tDNA-AT488 hybridization. (D) Linear correlation between obtained peak intensity on each aMZI sensor against its corresponding surface density collected from both BSA/BSA-biotin and aminated probe and negative control probe (pDNA-Am and npDNA-Am, respectively) approaches represented by rectangle and circle, respectively.

The fluorescence images of the aMZI sensors depict a pronounced fluorescence intensity emanating from the spiraled Si_3_N_4_ waveguide surface, contrasting with the SiO_2_ area (Figure 4A). This observation underscores the selectivity of the functionalization steps toward the sensing area on the chip surface, attributed to the material-selective, carboxylic acid-terminated coating applied onto the chip surface beforehand, allowing selective deposition of BSA on the sensor surface (see Materials and Methods). Next, we extracted the average fluorescence intensity cross-section by selecting a specific area in the fluorescence image (box in Figure 4A). The measured intensity profiles on all aMZIs (except the negative control aMZI-6) exhibited prominent peaks at the Si_3_N_4_ waveguide surface and minimal intensity elsewhere (Figure S3), indicative of pronounced hybridization selectivity within the aMZI sensing region. Notably, the peaks demonstrated exceptional homogeneity across the surfaces of the aMZI waveguides, displaying marginal intensity variations (Figure 4B). Hereafter, each aMZI sensor was subsequently associated with an average peak intensity, denoted as *I*_*green*_, and correlated with its signal response previously obtained from the aMZI readout (Figure 4C).

### Quantifying the Density of the Probe and Target DNA

**Figure 5:**
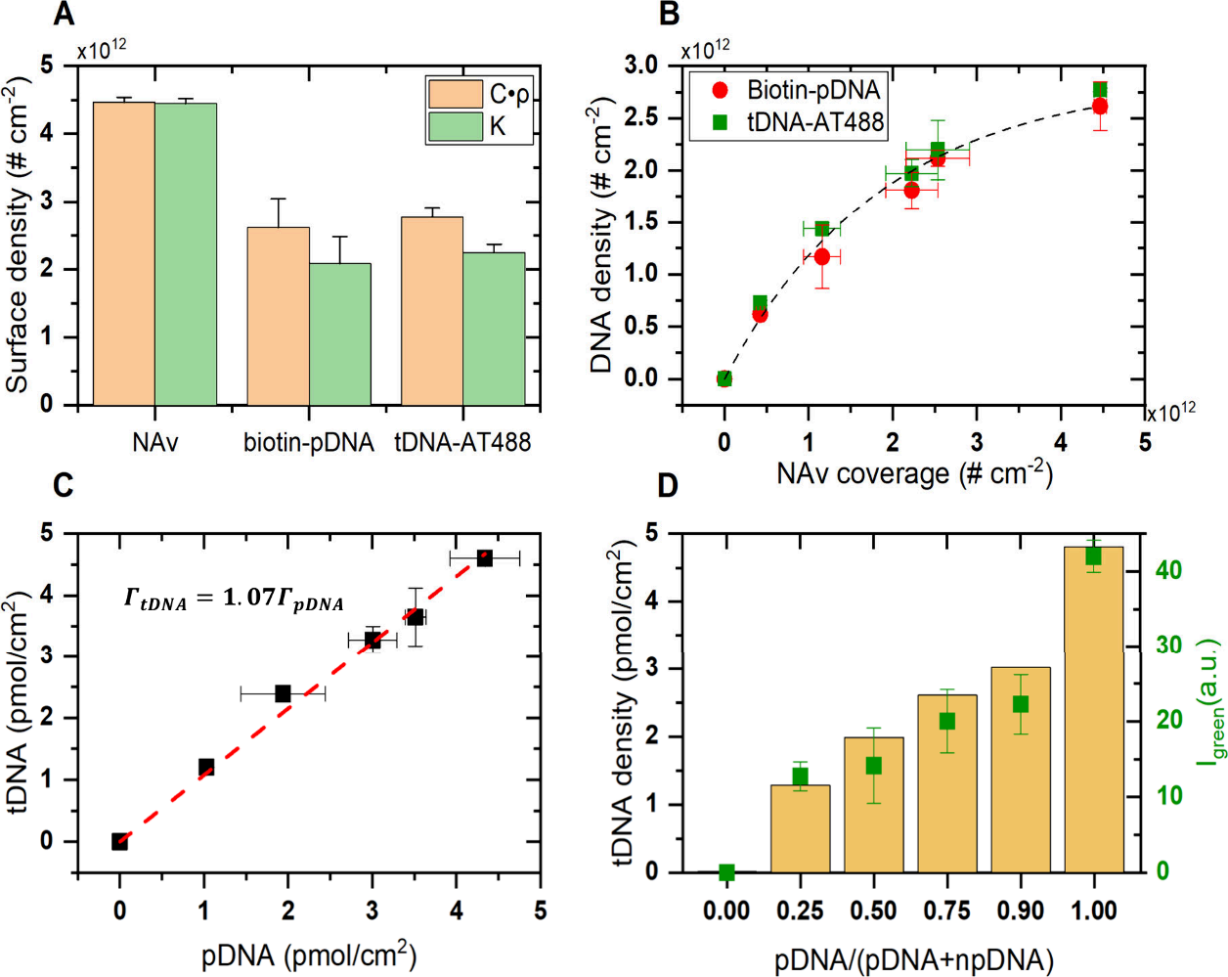
Quantification of the generated aMZI signal response (Δλ), (A) during immobilization of NAv, biotin-pDNA, and complementary tDNA using variable ρ (orange, eq 2, estimated using ρ = 1.35, 1.73, and 1.70 g/cm^3^ for NAv, ssDNA, and dsDNA, respectively) and fixed ρ=1.35 g/cm^3^ (green, eq 1), at full (100%) BSA-biotin coverage. (B) Densities of biotin-pDNA and hybridized tDNA as a function of the estimated NAv surface coverage obtained on each individual aMZI with the adjusted BSA/BSA-biotin; the dashed line serves as a guide to the eye. (C) Linear correlation of the immobilized biotin-pDNA and tDNA hybridization. (D) Surface density estimation of the hybridized tDNA-AT488 on the amino-pDNA/npDNA functionalized surface (orange bars) correlated with its fluorescence peak intensity (green dots) extracted from each aMZI surface.

To quantify the surface density of NAv and of the probe and target DNA, we used a method described before.^16^ The real-time signal shift obtained during the binding process of an adsorbing entity is used to quantify its surface coverage (Γ) using eq 1:^16,17^
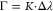
1where Δλ is the sensor response in picometer (pm) and *K* (0.029 ng pm^–1^ cm^–2^) is a constant dependent on the molecular mass density, the sensor’s bulk sensitivity of 2000 nm per refractive index unit (RIU) and the Cauchy extrapolation model (229 pm/nm).^15^ This equation and *K* value are valid for proteins with a bulk mass density (ρ) of 1.35 g/cm^3^, however, for different analytes different mass densities should be taken into account, using the linear relationship of molecular mass density and RI.^28,29^ For example, for the commonly used adsorbing polypeptide poly(l-lysine) ρ ∼ 1.18 g/cm^3^,^13^ which is significantly less than that of NAv (∼1.35 g/cm^3^).^17^ On the other hand, DNA has a slightly higher mass density ∼ 1.72 g/cm^3^.^30^ Therefore, an alternative calculation is used here in which a constant (*C*) independent of mass density was adopted, see eq 2:
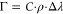
2Here, *C* is equal to 2.15 × 10^–11^ cm pm^–1^ for an aMZI at a bulk sensitivity of 2000 nm RIU^–1^. For the sake of comparison, we estimated the surface coverages using both eqs 1 and 2, as presented in Figure 5A. Accordingly, the BSA/BSA-biotin adjusted surface coverage presented in Figure 3, can now be quantified by using eq 2. The estimated surface coverage of NAv immobilization on each aMZI is plotted against its corresponding surface densities of biotin-pDNA and tDNA-AT488 (Figure 5B).

Notably, the measured maximal NAv coverage in our case is consistent with the theoretical estimated coverage for an NAv hexagonal close-packed (HCP) arrangement () on an ideal flat surface (4.6 × 10^12^ # cm^–2^), taking into account an avidin protein diameter of ∼ 5 nm and HCP of 0.9069.^31,32^ In terms of pDNA/tDNA (DNA) binding to the NAv layer, the initial slope depicted at Figure 5B indicates that a majority of potentially available two binding sites on each immobilized NAv (4.2 × 10^11^ # cm^–2^) are fully occupied by DNA (6.7 × 10^11^ # cm^–2^), leaving approximately 20% unoccupied. As the NAv coverage increases further, there is a gradual reduction in the amount of DNA occupying the NAv sites, eventually reaching 30% DNA occupation at the maximal NAv coverage. This hindrance may stem from hydration and/or electrostatic forces induced by negatively charged probes in close proximity to each other by positioning them onto neighboring NAv. This results in preventing or at least limiting the possibility of two biotin-pDNA binding onto a single NAv, which was also observed before.^33−35^ Moreover, this effect could become more pronounced during the hybridization with tDNA, where the hybridization efficiency could be affected by repulsion arising from the negatively charged immobilized probes, the fraction of tDNA hybridized on the surface, and the charge interaction between the surface interface and free tDNA in the bulk.

Regarding the DNA hybridization efficiency, there are slight fluctuations between low and high pDNA coverages. Nevertheless, a linear correlation between the amount of probe on the surface and the hybridized target is observed, as seen in Figure 5C. Thus, on average the available probes on the surface hybridize fully to their complementary strands. Hence, the decreasing trend in the immobilization efficiency of the probe has no further effect on the hybridization efficiencies here. Our findings align with previous studies, which reported 100% hybridization efficiency at lower probe DNA densities (<3 × 10^12^ #/cm^2^), whereas higher densities (>5 × 10^12^ #/cm^2^) led to reduced efficiency.^36^ Various approaches have been explored to improve hybridization efficiency, such as modifying probe linkers and optimizing surface functionalization.^37,38^ In our case, the surface roughness introduced by the carboxylic acid layer, along with BSA scaffolding and NAv functionalization, increases probe spacing, effectively minimizing steric hindrance and electrostatic interactions.

**Figure 6:**
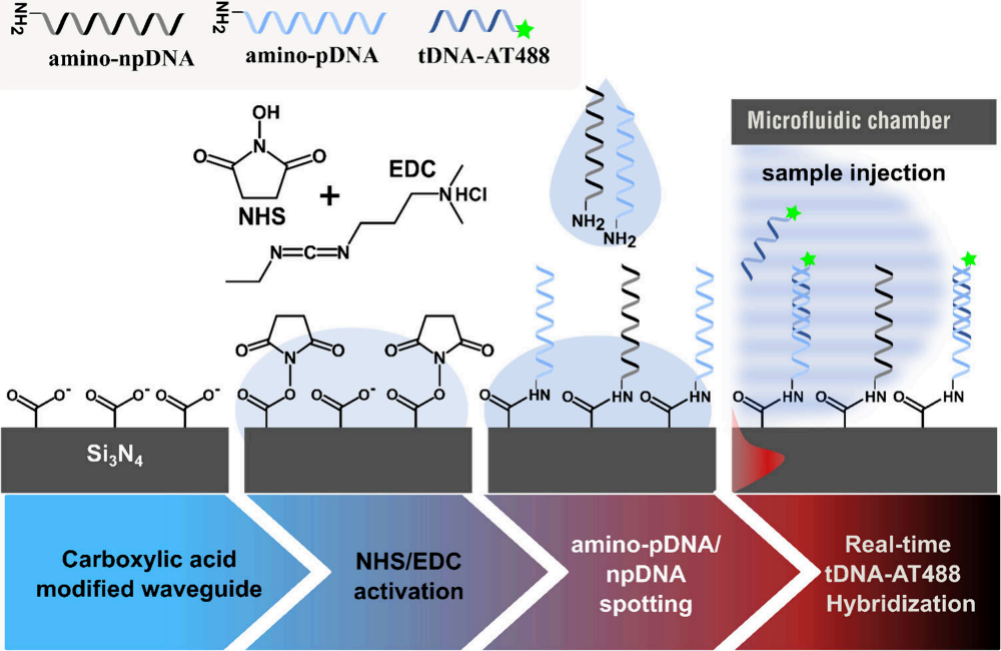
Surface functionalization using aminated ssDNA probes attached covalently onto NHS-activated carboxylic acid groups on the surface. To obtain a controllable surface coverage, two strands with distinct sequences (one probe, one nonprobe) are mixed at varying ratios, followed by spotted onto the aMZI surface.

Besides the aforementioned surface functionalization method, an alternative approach was attempted by directly spotting a mixture of an aminated ssDNA probe (amino-pDNA) and a nonprobe ssDNA sequence (amino-npDNA, negative control) onto the NHS-activated carboxylate aMZI surface, instead of using BSA/BSA-biotin/NAv/biotin-DNA chemistry (Figure 6). To control the surface density, amino-pDNA was mixed with a nonprobe ssDNA sequence (amino-npDNA), which was introduced as a dummy, to achieve the desired mixed percentages. Using the Scienion dispenser, each aMZI sensor was spotted and incubated with a prepared mixture of amino-pDNA and amino-npDNA (Figure S1). A limitation of this functionalization method was the inability to monitor the binding of probe DNA in real-time due to *ex-situ* incubation. However, this approach led to increased selectivity, specificity, and the signal-to-noise ratio. Moreover, this route reduced the amount of fabrication steps and provides a fully covalent, protein-free route which is advantageous for final assay applications.

**Figure 7:**
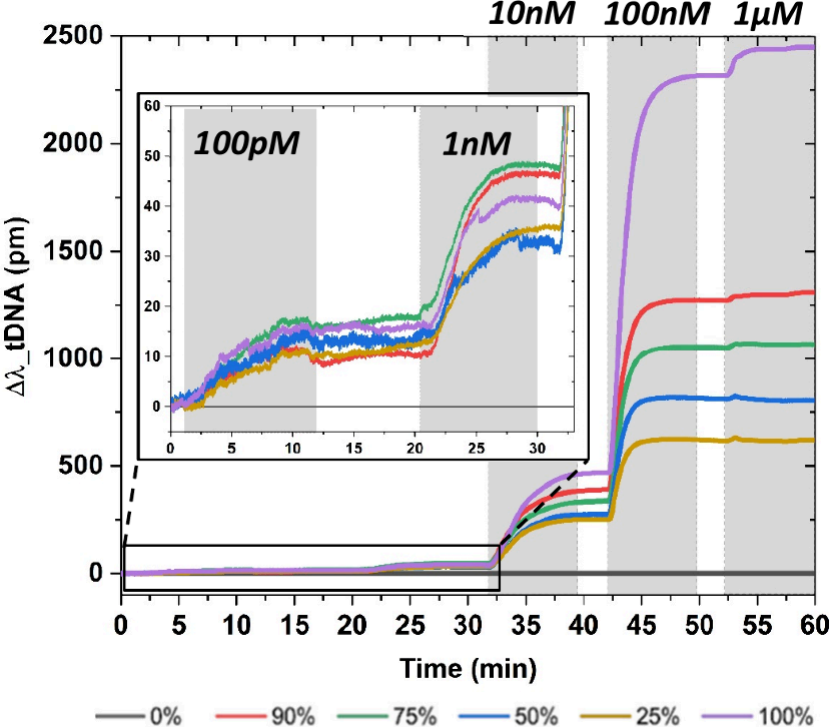
Real-time hybridization on the aMZI surface functionalized using amino-functionalized ssDNA probes (amino-pDNA) attached covalently on NHS ester-treated surfaces containing carboxylic acid groups. The amino-pDNA is complementary to the tDNA-AT488, while the other, nonprobe strand (amino-npDNA) is used as a reference. To control the surface coverage, the two strands (amino-pDNA and amino-npDNA) were mixed at varying ratios and then spotted on a different sensor area of the aMZI surface; each sensor is depicted as a colored line (percentages indicate the fraction of pDNA).

After an incubation period, a stable ssDNA biorecognition layer was formed without the need for an intermediate scaffold layer (BSA/BSA-biotin/NAv). Subsequently, a real-time signal response during hybridization with tDNA-AT488 was directly obtained (Figure 7). A hybridized signal response as low as 100 pM is measured on the aMZI sensor, demonstrating the potential of aMZI technology for diagnostic purposes. At such low tDNA-AT488 concentrations, the hybridization appears to be independent of the surface coverage and became more noticeable at higher tDNA concentrations (Figure 7), resembling previous data obtained using the BSA/BSA-biotin approach (Figure 3F). This direct covalent approach significantly reduces complexity and enhances surface controllability. However, the removal of the intermediate layer complicates the quantification of each functionalization step. Therefore, the initial method was primarily used for quantification purposes (Figure 2), as it provides more detailed insights into the binding process at each functionalization step.

The analysis above facilitates the quantification of signal responses generated due to DNA absorption without the need for an intermediate protein layer or predetermined surface coverage. Additionally, since the complementary strand hybridizes fully (1:1) to its probe on the surface, the signal response obtained due to hybridization of target ssDNA can now be utilized to indirectly determine the pDNA surface coverage. Therefore, we utilized eq 2 to predict the surface coverage of amino-pDNA on an amino-pDNA/npDNA mixed surface approach described previously (Figure 6). In this scenario, the spotting of varying ratios of amino-pDNA/npDNA follows a more predictable (linear) surface functionalization compared to the BSA/BSA-biotin approach, as shown in (Figure 5D). For instance, the hybridization of tDNA-AT488 on 25% amino-pDNA surface coverage shows the lowest surface density (∼1.1 pmol cm^–2^), whereas at 100%, the estimated surface density is four times higher (4.5 pmol cm^–2^). This indicates an increase of 1 pmol cm^–2^ for every 25% increase in surface coverage (or mixing ratio). A higher probe density would typically be expected for 100% amino-pDNA, as seen in self-assembled monolayers using thiolated probes.^36,37^ However, in this case, the inefficiency of NHS/EDC activation and amine coupling leads to incomplete functionalization.^39,40^ Additionally, the roughness of the carboxylic acid layer creates larger gaps between probes, further contributing to reducing electrostatic repulsion. These combined effects facilitate 1:1 hybridization.

After the signal response was successfully quantified, the obtained surface coverage for the BSA/BSA-biotin and amino-pDNA/npDNA (Figure 5D) is subsequently associated with the average peak intensity, denoted as *I*_*green*_. Hereafter the fluorescence intensity is correlated with its obtained surface density (Figure 4D). The correlation observed between the fluorescence peak intensity and the tDNA surface density yielded a simple linear relationship (Γ*_tDNA_* = Γ*_p__DNA_* = 0.1*I_green_*). This correlation significantly enriches the analytical toolbox for estimating DNA surface density based on fluorescence data, thereby offering a noteworthy extension of existing methodologies. However, this relation will need to be recalibrated for each individual fluorescence microscope using the method described in this work and holds only with fixed microscope settings. Yet, the linearity provides an independent verification of the sensor response and can be used to compare different immobilization strategies. Together, these quantification methods provide a comprehensive and cross-validated measurement of the molecular density. While the optical signal method offers absolute quantification based on RI changes,^8,13,14^ the fluorescence-based technique provides a relative assessment, allowing for internal consistency and comparative analysis. By integrating these two complementary methods, we enhance the reliability of our results and ensure their alignment with the established literature, reinforcing the validity of our findings.

## Conclusions

In the present work, we successfully introduced an approach for controlling DNA probe density by utilizing the BSA/BSA-biotin mixing ratio with subsequently adsorbed NAv as a platform on the sensor surface for subsequent probe immobilization. This approach allowed the quantification of the NAv density at various biotin-BSA coverages and the consecutive DNA-based recognition layer surface density on the aMZI biosensor. The stoichiometric ratio at successively higher neutravidin coverages showed a decrease in the efficiency of DNA bound to the neutravidin binding sites. This comprehensive analysis allowed us to correlate the obtained DNA surface densities with the fluorescence intensities observed over the aMZI sensors following the hybridization of fluorescently labeled complementary strands. Using covalent surface functionalization via the amide coupling reaction offers a simpler and more direct method for controlling the surface density. The fluorescence intensity analysis in this scenario allows for the estimation of the aminated probe DNA on the surface. These findings highlight the efficacy of our methodology in controlling and quantifying surface coverage, thereby enhancing the understanding and optimization of biosensor performance.

## Materials and Methods

BSA and biotinylated BSA were purchased from Thermo Scientific and dissolved at a concentration of 2 mg/mL of 20% Trehalose in 50 mM MES. NHS and EDC were purchased from Sigma-Aldrich, and both components were dissolved to concentrations of 0.1 and 0.2 M, respectively, in 10 mM MES (5.5 pH), right before surface activation. Neutravidin was purchased from Thermo Scientific and diluted in 1 mM NaOH to a concentration of 1 mg/mL. The aMZI chips were purchased from Lionix B.V. and treated with a material-selective coating by Surfix Diagnostics B.V. Lyophilized aminated probe (amino-npDNA) used as negative control sequence (5′-TTTTTTTTTT/3AmMO/-3′), both biotinylated and aminated-pDNA {5′-CTTGTTCTTTCTCTTCCTCGCCTGC/3BioTEG/for biotin or/3-amino-MO/-3′ for amine}, and its Alexa488-labeled complementary strand {5′-GCAGGCGAGGAAGAGAAAGAACAAGAAAAAGAG-AAGGATGCAGAAAACAA/3ATTO488N/-3′} were ordered from IDT and dissolved in TE buffer (TE = 10 mM TRIS + 0.1 mM EDTA) to a concentration of 100 μM then stored in −20 °C. For spotting, a phosphate buffer (PB) was prepared by mixing 4.82 g of dibasic and 1.52 g of monobasic sodium phosphate to a final phosphate concentration of 1.5 M (7.4 pH). All buffers and chemicals used were purchased from Sigma-Aldrich unless otherwise stated.

### aMZI Data Acquisition and Fluidic Setup

The aMZI sensor array comprises six individual aMZI sensors, each measuring 650 μm × 400 μm, and fabricated using single-stripe silicon nitride planar waveguide technology (TriPleX) with a stoichiometric Si_3_N_4_ core. In each sensor, the exposed section of the Si_3_N_4_ waveguide within the sensing arm is 12.5 mm in length, 1000 nm in width, and 100 nm in height, situated atop a 6 μm SiO_2_ substrate.^11,12,18^ During the real-time measurements, light with a fixed resonance wavelength of 850 nm is supplied to the biosensor via a VCSEL laser source and allowed to propagate through the waveguide. The waveguide is divided into two equal propagating spiral-shaped waveguide sections denoted as the “reference arm and the “sensing arm” as depicted in Figure 1B. The propagating beam at the reference arm is protected by a layer of SiO_2_; in contrast, in the sensor arm the SiO_2_ layer is etched to expose the Si_3_N_4_ waveguide, thus allowing the waveguide to interact with the environment. A fraction of the confined light within the exposed waveguide section extends into the surrounding medium as an evanescent field, which propagates along the waveguide surface and exhibits an exponential decay with distance from the Si_3_N_4_ interface. When a target molecule binds to the sensor surface, it induces changes in the refractive index (RI) of the sensor arm relative to the reference arm. Recombination of the light of the two arms leads to interference and a resulting phase shift (Δλ) of the input light (in pm).

During sample injection on BSA/BSA-biotin coated aMZI sensors, a fluidic chamber made of Teflon was clamped on top of the biosensor chip (Figure 1C), and a buffer solution (running buffer) was continuously introduced onto the chip surface and allowed to stabilize. In case of NAv functionalization, a 10 mM MES (pH 5.5) buffer was used as a running buffer, then replaced by PBS during the biotin-pDNA binding, and finally for tDNA hybridization 4xSSC buffer was selected as a running buffer. Prior to tDNA hybridization, the aMZI chip mount (Figure 1C) was placed in a laboratory oven (VWR INCU-Line IL 10) and preheated to 40 °C for 1 h. Similar conditions were used for the hybridization of tDNA-AT488 on the alternative (amino-pDNA/amino-npDNA) approach.

The experimental setup incorporates an automated switchable valve system to streamline the loading of 100 μL plug samples into the fluidic system (Figure S4). Following loading, the plug is introduced into the fluidic chamber via a syringe pump filled with the designated running buffer (see Supporting Information for more details). This method ensures consistent, seamless, and stable transitions from the sample to running buffer with each injection, thereby enhancing reproducibility. Notably, within this fluidic framework, axial mixing of the sample with the running buffer is anticipated, particularly at the plug’s termination point, leading to the establishment of a concentration gradient. Therefore, the injections are delineated as a gradient area’s in Figure 3A. Furthermore, the flow rate for all running buffer and sample injections was set at 20 μL/min, except during the NAv injection, where the flow rate initially commenced at a slower rate (4 μL/min for 3 min) before returning to 20 μL/min. This adjustment was implemented to prevent the initially generated rapid binding shifts of NAv on the surface, which could potentially disrupt and become faster than the sampling rate of the software.

### BSA/BSA-Biotin Mixing Ratio Spotting

The surface of the aMZI sensors, commercially treated with a material-selective coating provided by Surfix Diagnostics B.V., provides a thin layer with exposed carboxylic acid groups (COOH) on the Si_3_N_4_ waveguides and a PEG antifouling group on the remaining SiO_2_ surface area (the nonsensing area). Consequently, the sensing areas can be selectively modified with a biorecognition layer by addressing the carboxylic acid groups of the primer layer. This was achieved by incubating a mixture of 0.1 M NHS and 0.2 M EDC in 10 mM MES (5.5 pH) manually for 15 min on the entire chip surface. The NHS/EDC activates the COOH waveguide surface with an amine-reactive NHS-ester. Immediately after the NHS/EDC activation, the surface was rinsed with 10 mM MES and PBS and then placed in a spotter (Scienion S3) for the follow-up incubation process. Hereafter, a mixed ratio of BSA/BSA-biotin (or amino-pDNA/amino-npDNA for the second approach) was prepared, precisely dispensed in the sensor well, and allowed to incubate for 2 h inside a humidity-controlled chamber. The incubated aMZI chips were thoroughly rinsed with MQ.

The mixture of BSA and BSA-biotin or amino-pDNA and amino-npDNA was prepared to a concentration of 10 μM in 10 mM MES with 3% trehalose and 1.5 M PB, respectively. Pretreated chips with material-selective coatings were activated using 0.1 M NHS/EDC in MES buffer at pH 5.5 for 15 min at room temperature. Excess reagents on the chip surface were washed off with 10 mM MES and PBS, and the chips were placed in the spotting chamber under 60% humidity at room temperature (∼22 °C).

Subsequently, the spotting protocol was initiated using a Scienion dispenser connected to a designated nozzle. The protocol automatically dispensed 27 nL of the BSA/BSA-biotin or amino-pDNA/amino-npDNA mixture onto the aMZI sensor. The nozzle was then thoroughly rinsed with MQ and SciClean. This process was repeated for each sensor well with different mixture ratios as illustrated in Figure S1 (see also Table S1). After an incubation period of approximately 1 h, the chip was carefully submerged in PBS with 7% BSA or a 5 mM solution or amino-PEG10 for 30 min to block any remaining active (unoccupied) sites on the BSA or aminated ssDNA surface, respectively. Thereafter, the surface was washed with PBS containing 0.05% Tween20 and PBS with 3% trehalose. Finally, the chip was stored in a vial filled with 10 mM EDTA in PBS until further use.

### Fluorescence Intensity Assessment

Fluorescence microscopic analysis was performed using an IX71 Epi-fluorescence microscope (Carl Zeiss AG, Germany) equipped with a 10x objective For the AT488 fluorescent labeled tDNA excitation, a filter cube of 460–490 nm was used, and the emission was collected using a 520 nm emission filter. Images were captured with a digital camera, an Olympus DP70 CCD camera, at a resolution of 1092 × 1080 pixels. Microscope settings of 100% illumination intensity and 1-s exposure time were fixed for all acquired images. Additional details regarding image analysis and processing procedures are provided in the Supporting Information.

## Supporting Information Available

Mixing ratios layout on aMZI sensors; experimental procedures; unmodified signal response; fluorescence microscopy images; microfluidics system setup (PDF)
