# Supplementary material for: Quantifying and Controlling DNA Probe Density on the Surface of Silicon Nitride Optical Waveguides
Source: Langmuir. 2025 Apr 23;41(17):11205–14. doi: 10.1021/acs.langmuir.5c01064 (PMC12060268; doi:10.1021/acs.langmuir.5c01064)
Supplement: Supplementary file 1 — la5c01064_si_001.pdf [file la5c01064_si_001.pdf]

Supporting information

Quantifying and controlling DNA probe density on the surface of silicon nitride optical waveguides

Samer Aphrham[a,b], Mark Verheijden[b*], Jurriaan Huskens[a*]

[a] Department of Molecules and Materials, Faculty of Science & Technology, MESA+ Institute and TechMed Centre, University of Twente, PO Box 217, 7500 AE Enschede, The Netherlands
[b] Qurin Diagnostics B.V., Emmy Noetherweg 2, 2333 BK Leiden, The Netherlands

Table of Contents:

Table S1: Mixing ratios layout on aMZI sensors S2

Figure S1: Experimental procedures S2
- mixing ratio and spotting layout

Figure S2: Unmodified signal response S3
- Raw signal response during biotin-pDNA and tDNA-AT488 injections
  Bulk effects and signal shifts in negative control aMZI sensor

Figure S3: Fluorescence microscopy images S4
- aMZI sensor fluorescence cross-section intensity profile

Figure S4: Microfluidics System Setup...S5
- Plug injection and Axial mixing

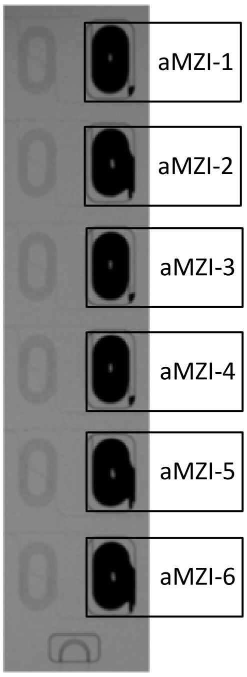

Table S1: mixed fraction prepared and spotted on each sensor for biotinylated BSA and aminated ssDNA approach

**Sensor**	**BSA/BSA-biotin**	**amino-pDNA/amino-npDNA**
aMZI-1	0% BSA-biotin+100% BSA	0% pDNA+100% npDNA
aMZI-2	1% BSA-biotin+99% BSA	90% pDNA+10% npDNA
aMZI-3	2.5% BSA-biotin+97.5% BSA	75% pDNA+25% npDNA
aMZI-4	5% BSA-biotin+95% BSA	50% pDNA+50% npDNA
aMZI-5	10% BSA-biotin+90% BSA	25% pDNA+75% npDNA
aMZI-6	100% BSA-biotin+0% BSA	100% pDNA+0% npDNA

Figure S1: Brightfield image of aMZI sensors after spotting process and incubation for 1 h. The spotting was performed on the right side of the sensor as the left side is protected with SiO_2 to maintain the propagating light. Each sensor was spotted with a mixed fraction of either BSA/BSA-biotin or amino-pDNA/amino-npDNA to a final concentration of 10 µM in 1.5 PB as shown in Table 1.

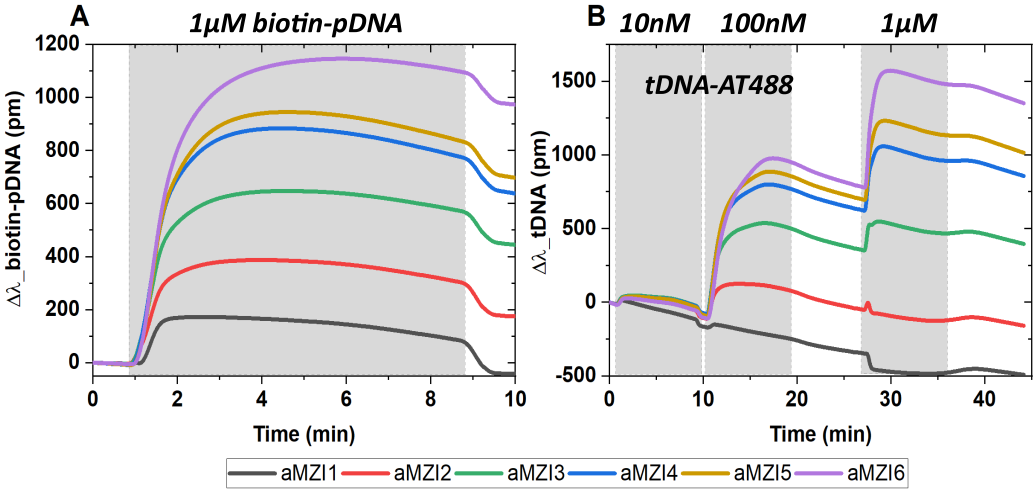

Figure S2: Unmodified real-time signal response during functionalization of (A) biotin-pDNA followed with (B) the hybridization with tDNA-AT488 on immobilized NAv protein using BSA/BSA-biotin coated surface.

Figure S2 shows the raw (real-time) signal response during the injection of 1 µM biotin-pDNA (Figure S2A) and 10 nM to 1 µM tDNA-AT488 (Figure S2B). A signal shift is observed on all aMZI sensors, including the negative control sensor (aMZI-1), during the transition from the running buffer to the sample injection. The signal response detected on aMZI-1 is attributable to bulk changes on the surface caused by additives in the sample stock solutions (such as ionic strength and pH variations). When the running buffer is reintroduced to the chip surface, these bulk effects gradually diminish. For aMZI-1, the signal returns to baseline levels, whereas the other aMZI's display a net signal shift due to the mass bound to the aMZI surface. Additionally, an intrinsic linear signal drift is observed, originating from external sources such as the fluidic chamber clamp system and/or electronic components. Given these effects on the signal response, it is crucial to include a negative control on the chip to account for and subtract these non-specific effects from the binding signal.

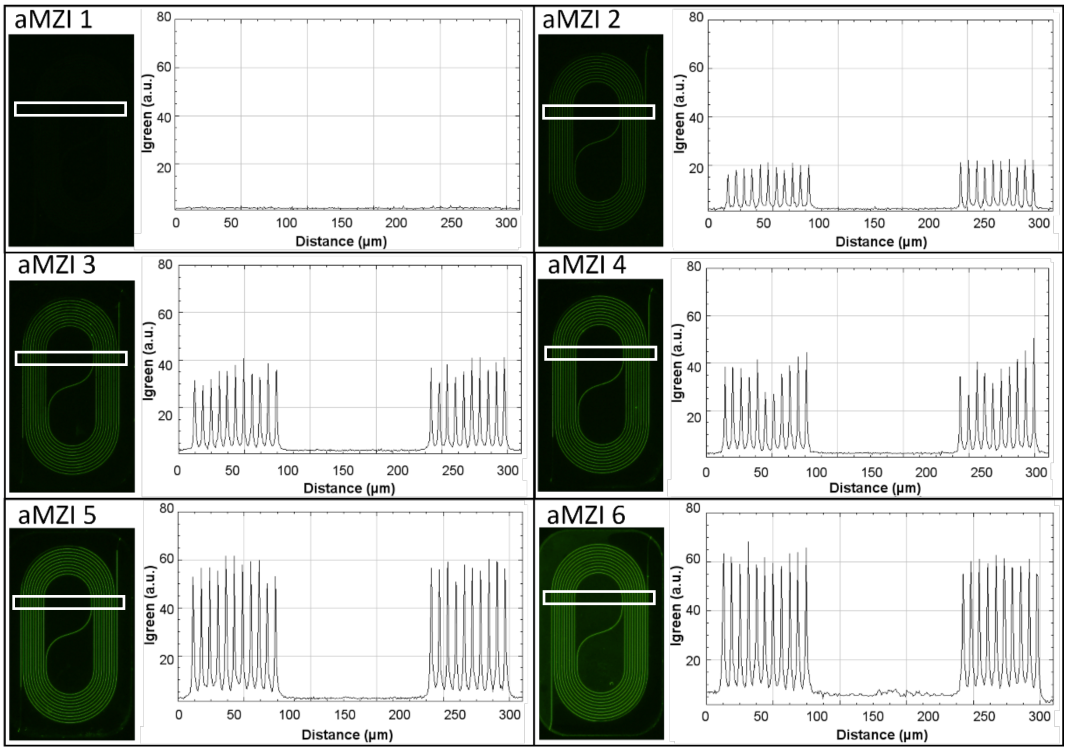

Figure S3: Unmodified aMZI sensors fluorescence images and its corresponding average intensity profile taken after saturation with 100 nM tDNA-AT488. The average intensity profile region is indicated by white rectangle.

For the intensity profile characterization, we first acquired the average cross-section profile intensity by selecting a certain area on the image using ImageJ software. The obtained intensity profile was then uploaded in Origin2023 and the multipeak fitting tool was utilized. Then systematically, the data underwent fitting, and the baseline graph was carefully subtracted for the extraction of maximal peak intensities. In total 21 peaks were found for each sensor signifying the amount of waveguide winding on a single aMZI sensor. A subsequent calculation yielded the average of all peaks. This analysis was replicated for the entire ensemble of aMZI's. Each individual aMZI sensor average peak intensity was then plotted against its estimated surface density, and a linear fit was obtained from all data points.

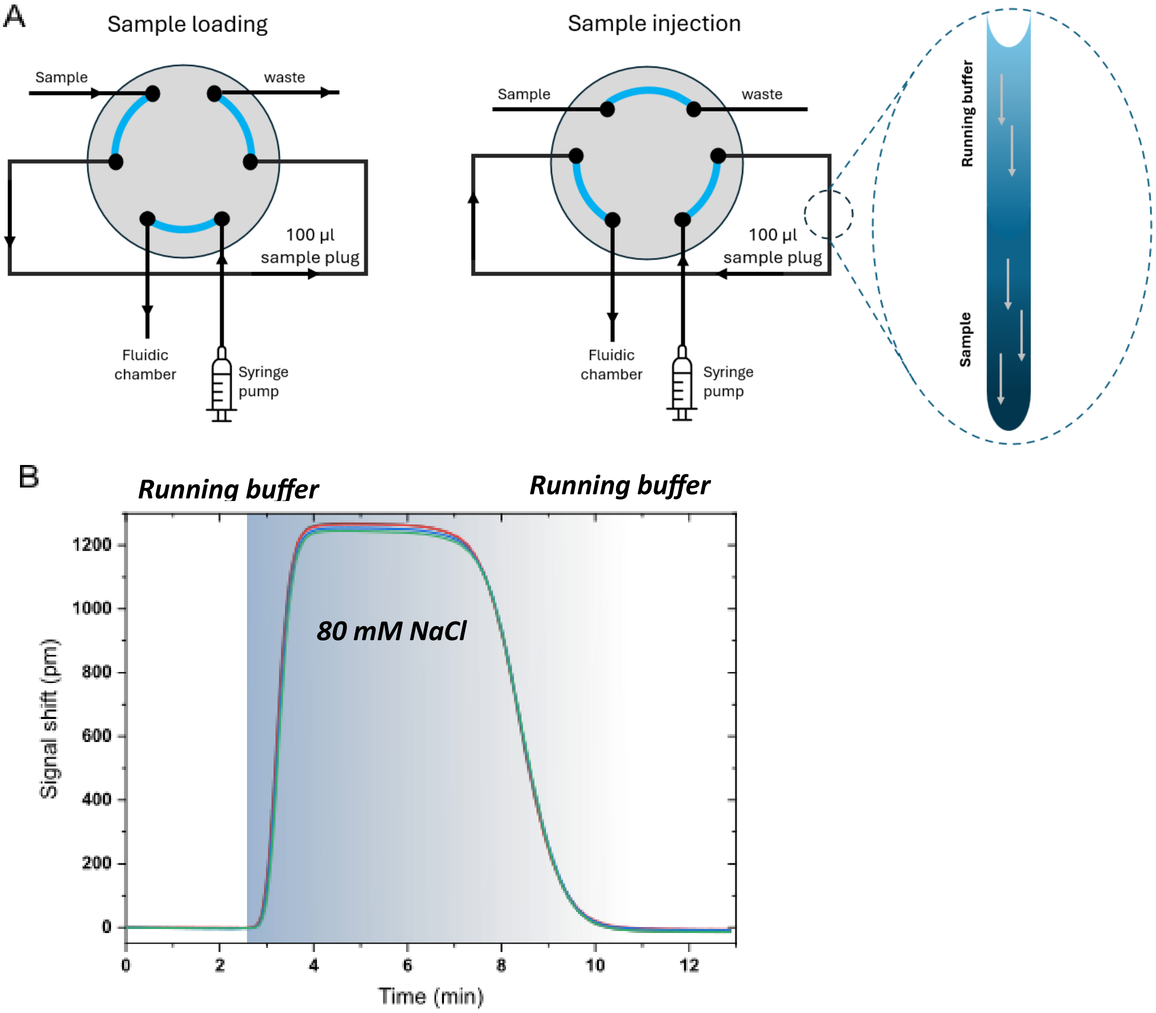

Figure S4: illustration of the microfluidics system showing A) the valves setup during sample loading and sample injection, in which at the end of the sample plug the axial mixing is illustrated as parabolic flow profile and B) measured in real-time by injecting 80mM NaCl in running buffer (PBS).

The injection was performed using an automatic valve system to load and inject a 100 µl sample plug into the fluidic chamber. To automate the injections and control the flow rate, the fluidic system was managed by a script. In the sample loading configuration, the running buffer is injected directly onto the chip, while simultaneously the sample is withdrawn into a 100 µl plug using a peristaltic pump. Subsequently, the valve is switched to the sample injection configuration, where the loaded sample is injected into the fluidic chamber using the syringe pump (Figure S5A). At the end of the plug, axial mixing occurs due to drag on the tubing wall.[1] To demonstrate axial mixing, we injected 80 mM NaCl in PBS (running buffer) at a flow rate of 20 µl/min, as shown in Figure S5B. When the sample reaches the chip surface (~3 min), a steep increase in signal shift is measured due to the change in bulk refractive index. As the running buffer is reintroduction at the end of the sample plug (~7 min), a gradual and less steep decrease in signal shift is observed.

References

1. Ruzicka, J. (Jarda) & Chocholouš, P. Next generation of flow analysis is based on flow programming. *Talanta* **269**, 125410 (2024).
